# Screening for Drought Tolerance in Maize (*Zea mays* L.) Germplasm Using Germination and Seedling Traits under Simulated Drought Conditions

**DOI:** 10.3390/plants9050565

**Published:** 2020-04-29

**Authors:** Abdelfattah Badr, Hanaa H. El-Shazly, Rasha A. Tarawneh, Andreas Börner

**Affiliations:** 1 Botany and Microbiology Department, Faculty of Science, Helwan University, Cairo 11790, Egypt; 2 Biological and Geological Sciences Department, Faculty of Education, Ain Shams University, Cairo 11341, Egypt; hanaahegazy@edu.asu.edu.eg; 3 Gene Bank Department, Leibniz Institute of Plant Genetics and Crop Plant Research (IPK) Corrensstr. 3, D-06466 Seeland, OT Gatersleben, Germany; tarawneh@ipk-gatersleben.de

**Keywords:** maize, abiotic stress, germination, seedlings, heritability, climate change

## Abstract

Maize is known to be susceptible to drought stress, which negatively affects vegetative growth and biomass production, as well as the formation of reproductive organs and yield parameters. In this study, 27 responsive traits of germination (G) and seedlings growth were evaluated for 40 accessions of the Leibniz Institute of Plant Genetics and Crop Plant Research (IPK) germplasm collection, under no stress and simulated drought stress treatments by 10%, 15%, and 20% of polyethylene glycol (PEG). The three treatments significantly reduced G% and retarded seedlings growth, particularly the 15% and 20% PEG treatments; these two treatments also resulted in a significant increase of abnormal seedlings (AS). The heritability (*H*^2^) and correlations of the traits were estimated, and drought tolerance indices (DTIs) were calculated for traits and accessions. The *H*^2^ of G% values were reduced, and *H*^2^ for AS% increased as the PEG stress increased. Positive correlations were found between most trait pairs, particularly shoot and root traits, with 48 highly significant correlations under no stress and 25 highly significant correlations under the 10% PEG treatments, particularly for shoot and root traits. The medium to high heritability of shoot and root seedling traits provides a sound basis for further genetic analyses. PCA analysis clearly grouped accessions with high DTIs together and the accessions with low DTIs together, indicating that the DTI indicates the stress tolerance level of maize germplasm. However, the resemblance in DTI values does not clearly reflect the origin or taxonomic assignments to subspecies and varieties of the examined accessions.

## 1. Introduction

Plants are occasionally exposed to a changing adverse biotic and/or abiotic factors, which may prevent plants from performing their maximum potential performance and can threaten their survival [1]. Drought is a primary abiotic constraint affecting crop production worldwide, due to shortages of fresh water. Drought stress on plants occurs when the available water lags continuous plant loss of water by transpiration [2]. With the weather expected to become generally drier and warmer, the situation may be further exacerbated as competition for water intensifies between people and crops [3]. Global climatic change will reduce the productivity of the most valuable crops and induce a detrimental impact on the ecological fitness of cultivated crops [4]. Webber et al. [5] predicted that climate change would lead to yield losses of maize and winter wheat, but drought stress would be more intensive for maize. In low-yielding years, drought stress persisted as the main driver of losses for both crops, with the elevated CO_2_ offering no yield benefit [5]. Maintaining crop productivity for future generations can be achieved by developing crop varieties tolerant to drought and heat from plant genetic resources [4].

Plant genetic resources (PGR) are plant materials of value for present and future generations of people. The PGRs have been, for a long time, recognized as indispensable sources of genotypic variation required for future breeding of new crop varieties [6]. In the last few decades, huge efforts were made to organize, store, and analyze all data gathered during exploration and collection missions [7]. The second Food and Agricultural Organization (FAO) report (2010) laid down bases for updating the global plan of action for the conservation and sustainable utilization of PGR. However, in the huge collections of PGR available in hundreds of gene banks around the world, only a little information is available on the extent of genetic variation in the traits of juvenile plant material such as germination rate and seedlings morphology traits in response to abiotic and biotic stresses. The Federal Ex-situ Gene Bank of the Leibniz Institute of Plant Genetics and Crop Plant Research (IPK) in Gatersleben is one of the largest gene banks in the world. The genetic diversity of wheat and barley accessions at the IPK recently received extensive screening for drought tolerance [8,9,10].

Maize (*Zea mays* L.) is ranked third to wheat and rice in the world’s production of cereal crops [11]. It is widely grown throughout the world in a wide range of agro-ecological environments. Being a C4 species, maize utilizes moisture and sunlight efficiently to produce high yield and total dry matter [12]. The demand for global maize production is increasing as a source of food, forage, oil, and biofuel, for the ever-increasing world human population. However, the annual maize yield loss due to drought was estimated to be about 15% of potential yield on a global basis [13,14]. Major maize producing areas will become warmer, drier, and subject to an array of new maize diseases and pests under climate change that may lead to alarming impacts on maize production under the hotter climate scenarios, although the degree of the impact varies across sites and rainfall pattern change [15]. This scenario calls for incorporating drought and heat tolerance traits into maize germplasm to offset predicted yield losses and sustain maize productivity in vulnerable sites [16].

The seedling stage of maize is especially sensitive to drought stress; it requires less water than the later vegetative and reproductive stages, but drought stress will influence their adaptation at the early crop establishment phase compared to the flowering and the longer anthesis-silk interval [17,18]. Maize seedlings emerge within 4–9 days after planting, depending on the intrinsic factors in the seed and environmental conditions such as temperature and moisture. The seedling stage of maize starts immediately after the emergence (VE) stage until the 5-leaf (V5) stage [12]. At this stage, the plants are very sensitive to environmental stress, such as drought, and severe stress at this stage results in total crop failure [19,20]. Selection indices based on secondary root traits along with grain yield parameters could lead to an increase in selection efficiency for grain yield under N stress condition [8].

Measures of drought tolerance based on germination and seedlings traits under controlled conditions and drought stress have been used by a few authors to identify candidate drought-tolerant genotypes [21,22,23,24]. In addition, drought sensitivity indices based on the response of seedling traits under stress conditions compared to the control have been recently applied to evaluate maize drought tolerance [25,26,27]. In wheat, root length, fresh weight, dry weight, cell membrane thermo-stability, and chlorophyll b content were positively correlated among themselves under both normal and stress conditions, whereas, shoot length was non-significant and negatively associated with all other studied characters except RWC [27]. In barley, significant negative correlations were found between G% and fresh weight with root length and shoot length under control conditions but only with fresh weight under drought stress [9]. Drought susceptibility index (DSI), stress tolerance index (STI), and stress index (SI) were most useful to identify genotypes differing in their response to drought [25,28]. Principal component analysis, biplot, and clustering methods are also increasingly used for comparisons of drought tolerance in maize [29,30,31].

The rationale for this study is to screen a core collection of maize germplasm from the IPK Gene Bank for drought tolerance. We adapted the use of variation in germination and seedling traits and their heritability and correlation as convenient approaches to identify candidate drought-tolerant accessions. This approach saves the laborious and time-consuming selection under field conditions for the identification of high grain yield potential [14,17,32].

## 2. Material and Methods

Seed material representing 39 accessions of a core collection of maize (*Zea mays* L.) at the Leibniz Institute of Plant Genetics and Crop Plant Research (IPK) Gene Bank from different origins and one Egyptian accession cultivar from the Agricultural Research Center in Giza, Egypt, were used in this study (Table 1). The seeds of each accession were divided into four groups of 50–60 each: one group was used for control, and the other three groups were used for three drought stress treatments by exposure to PEG_6000_ at concentrations of 10%, 15%, and 20%. Seeds of the control and drought treatments were germinated according to the International Seed Testing Association (ISTA) protocol [33]. The seeds of each treatment were divided into three sets each of 17–20 seeds and seeds of each set were germinated in special blotting paper sheets (Ahlstrom Munnksjö, 25 cm high × 60 cm wide) moistened either with distilled water or with 10%, 15%, and 20% *m/v*, PEG 6000). Then, the sheets were rolled to separate seeds from each other and held in transparent plastic bags and incubated in a growth cabinet (ASECOS EN 1440-2) at 25 ± 2/20 °C ± 2 (day/night) at a relative humidity of 60% under 16 h light/8 h dark at a light density of 400 µmol photons m^−2^s^−1^.

Evaluation of germination was made after nine days from seed sowing for the control and the 10% PEG treatment. The emergence of radicles and plumule of seeds exposed to 15% and 20% PEG was slow and the evaluation of germination for seeds exposed to these two treatments was postponed for one week (16 days after sowing). Seeds that had minimum radicle length of 3 mm were counted as germinated. Abnormal seedlings (AS), which failed to develop healthy seedlings, including few albinos, were recorded, and their percentage was calculated for each set of seeds. Shoot and root length of five seedlings for each replicate of the control and the 10% PEG treatment, were measured, their fresh weight was determined and dried at 80 °C for 48 h for dry weight determination. The moisture of the blotting paper rolls was continuously monitored, and water or PEG solutions were added to control and stressed seedlings to keep the paper rolls wet, and the seedlings were left to grow further in the growth cabinet.

The shoot and root length and fresh and dry weights were calculated for the control seedlings and seedlings exposed to 10% and 15% PEG after 16 days of sowing. After 21 days of sowing, the 5th leaf of the control plants and plants exposed to 10% PEG was well developed. For these two treatments, the length and width of the 4th leaf were measured for thee plants of each accession and the relative water content (RWC) was determined using the equation RWC% = [(FM−DM)/(TM−DM)] × 100, where, FM, TM, and DM, are the fresh, turgid, and dry masses, respectively. Three leaf discs for each accession were cut and immediately weighed (FM), then saturated to turgidity by immersing in cold water overnight, briefly dried, and weighed (TM), and oven-dried at 80% for 24 h and weighed (DM).

Germination parameters were assessed according to the ISTA rules [33]. Seedling traits were evaluated after 9 days of sowing for control seedling and seedlings exposed to 10% of PEG and after 16 days of sowing for the control and the 10% and 15% treatments, After 21 days of sowing, leaf length, leaf width and leaf RWC for the control and the 10% PEG treatment were determined. Seedlings were regarded as abnormal when the radicle or plumule was deformed or colored and fail to grow to healthy seedlings after 16 days of germination. Albino seedlings were scored for three genotypes in control seedlings and seedlings exposed to PEG treatments (10 seedlings for Zea 3282, one for Zea 3325, one for Zea 3346, and 2 for IW 237). The percentage of abnormal seedlings (AS) to the total number of germinated seedlings was calculated. The descriptions of the seedling´s measurements are in Table 2.

### Data Analyses

Box and Whisker charts illustrating the variation of the G%, AS%, and seedling traits under control and drought stress treatments were constructed using Excel 2016 for Windows. In addition, drought tolerance indices (DTIs) were calculated for germination (G-DTI) as the ratio of germination percentage of seeds exposed to each of the PEG treatments compared to the germination percentage of the control seeds. Similar DTIs expressing the change in the root, shoot, and leaf traits were calculated, as described in Table 2. The top 10% accessions scoring best performance expressed as highest means of the examined traits AS% and the bottom 10% accessions scoring the least performance in these traits were determined using Excel 2016 for Windows under control and PEG treatments. 

Analysis of variance (ANOVA) was conducted to compare accessions and traits using GenStat Ver. 18 (VSN International, Hemel Hempstead, UK) for the germination and the abnormal seedlings data and the seedling shoot and root traits after 9 days for the control and the 10% PEG treatment, and after 16 days, for the control, and 10% and 15% PEG treatments. The 20% PEG treatment was excluded from the shoot and root data analysis because the germination and seedlings growth rates were too slow. The ANOVA analysis for the leaf measurements and the leaf water content was performed for the control plants and the plants exposed to 10% PEG treatment only after 21 days of sowing. The probability of significance in ANOVA (*p* < 0.05) was used to indicate significant differences among genotypes, treatments, and interaction effects. Means were separated according to the Fisher’s least significant difference (LSD) at 0.05 levels of probability.

Correlations of the studied traits of maize accessions under control and PEG stress treatments were calculated using the GenStat 18. The degree of significance was indicated as *p* 0.05, *p*, 0.01, or *p*, 0.001. Broad-sense heritability was calculated according to Hallauer et al. [34] as follows: *H*^2^ = σ2g / (σ2g + σ2g × t/e + σ2e/re), where *σ2g* is genotype variance; *σ2g × t* is the variance of the interaction genotype × treatment, *r* is the replicates, and *e* is the error.

The DTIs were used as variables to construct a principal component scatter diagram using the software PAST Version 3.22 based on the Paleontological Statistics software tht wa developed by Hammer et al. in 2001 [35]. The PCA is applied to assign the variables to genotypes and to classify accession based on their sensitivity or tolerance to drought stress. The PCA utilizes orthogonal transformation to convert a set of possibly correlated variables into a set of linearly uncorrelated variables called principal components. This transformation is defined in such a way that the first principal component has the largest possible variance. PCA is sensitive to the relative scaling of the original variables in the PCA scatter plotting visualization. Eigenvectors generated by PCA were used to rank the accessions for their drought tolerance [30]. The grand average of the DTIs of all traits was calculated and used as a measure for the drought tolerance of accessions. 

## 3. Results

### 3.1. Variation in Germination and Abnormal Seedlings Percentage

**Figure 1:**
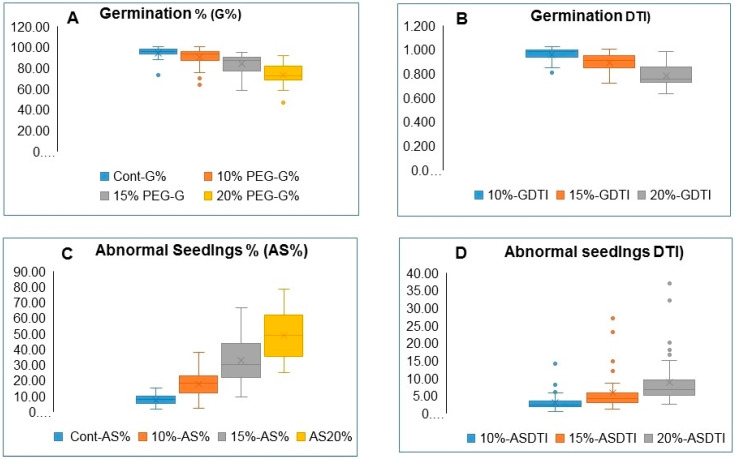
Box and whisker charts showing variation of G% and AS% for all maize accessions under control and 10%, 15%, and 20% PEG treatments: (**A**) and (**B**) boxplots illustrating reduction in G% and G%-DTI by the PEG treatments, respectively, (**C**) and (**D**) boxplots illustrating increase in the AS% and AS-DTI by PEG treatments, respectively. Full names of shoot traits and their DTIs are in Table 2.

The germination percentage (G%) of all accessions varied significantly, as indicated by the ANOVA analysis under both control and PEG stress treatments and showed significant reductions as the PEG concentration increased (Table 3). The box and whisker charts for the G% and G-DTIs (Figure 1A,B) illustrate substantial variations between accessions and treatments, as indicated by the lower and upper limits of the boxplots for each trait. The G% is less affected by the 10% PEG treatment as compared to the 15% and 20% PEG concentrations. The Zea 3244, the outlier accession in the control G% boxplot (Figure 1A), showed the lowest G% under the control and the PEG stress treatments (73.3%, 63.33%, 58.33%, and 46.67% for the control, and 10%, 15%, and 20% PEG treatments respectively). Other accessions that showed low G% under control and stress treatments were Zea 677, Zea 3324, and Zea 3244; the latter accession was the only outlier observed for the G% under the 20% PEG treatment. The sensitivity of germination to PEG treatments is clearly indicated by the reduction of G-DTI values as the PEG concentration increased from 10% to 15% and 20%, respectively (Figure 1B).

The mean AS% for all accessions showed a successive increase from a value of 7.41% under control conditions to 17.68% under 10% PEG, 32.44% under 15% PEG, and 48.68% under 20% PEG treatments, respectively (Figure 1C; Table 3). Significant differences (≤0.001) between accessions were recorded under both the control conditions and the PEG treatments. Unlike the drought tolerance indices (DTIs) of all other traits, the AS-DTI increased as the percentage of abnormal seedlings increased. It ranges from a low value of 0.67 for Zea 1224 to the highest value of 14.0 for Zea 1062. Three AS-DTIs of 14.0, 8.0, and 6.33 were scored as outliers for accessions Zea 1062, Zea 711, and Zea 3582, respectively, under 10% PEG. Four AS-DTIs of 27.0, 23.1, 14.8, and 12.0 were observed as outliers in the boxplot for AS-DTI of accessions Zea 711, Zea 1062, Zea 3576, and Zea 1102, respectively, under the 15% PEG treatment and five outliers were observed for AS-DTI values following exposure to 20% PEG, including the above-mentioned accessions plus Zea 323 (Figure 1D). The G%-DTI and AS-DTI values are given in Table A1.

### 3.2. Variation in Seedling’s Traits

**Figure 2:**
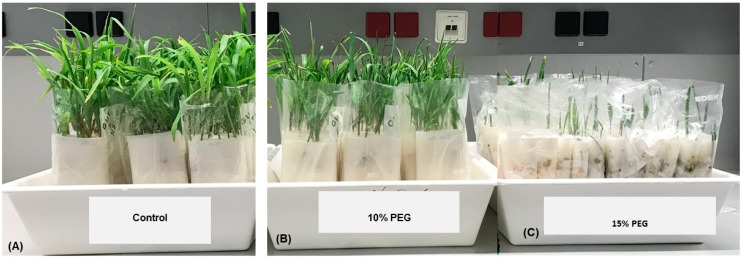
Photographs illustrating the impact of 10% and 15% PEG treatments on 21 days old seedlings of maize accessions. (**A**) control, (**B**) 10% PEG, (**C**) 15% PEG.

**Figure 3:**
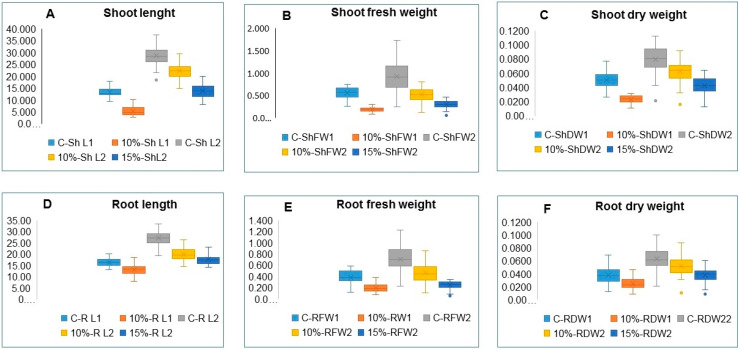
Box and whisker charts showing variation in seedling traits measured for control and the 10% PEG treatments after 9 days of sowing and for the control, and 10% and 15% PEG treatments after 16 days of sowing: (**A**) ShL, (**B**) ShFW, (**C**) SDW, (**D**) RL, (**E**) RFW, (**F**) RDW, respectively; 1 and 2 refers to measurements after 9 days and 16 days of seeds sowing.

The PEG treatments retarded the seedling growth of all accessions to the extent that it was not possible to evaluate variation in the seedling traits under the 20% PEG treatments. Figure 2 is a photograph showing the retardation of seedling’s growth by the 10% and 15% PEG treatments. Box and whisker charts show the variation in seedling traits for all accessions, measured for control seedlings and seedlings exposed to 10% PEG after nine days of sowing and for the control, and 10% and 15% PEG treatments after 16 days of sowing (Figure 3). The mean of the measured traits showed successive reductions as the PEG concentration increased at the two seedling stages of growth, i.e., 9 and 16 days after seed sowing. For the nine days old seedlings, the accessions revealed a highly significant variation (*p* ≤ 0.001) of the examined traits under control and 10% PEG treatment. Zea 3244. was an outlier in the 16-day-old control seedlings shoot length (C-ShL2) in Figure 3A and shoot dry weight (C-ShDW2) in Figure 2C. In seedlings exposed to 10% PEG, the same accession was the outlier for the shoot dry weight (10%-ShDW2) and root dry weight (10%-RDW2) in Figure 3C,F. The Zea 3244 was also an outlier in seedlings exposed to 15% PEG for shoot dry weight (15%-ShDW2) root fresh weight (15%-RFW2), and root dry weight (15%-RDW2) in Figure 3B,D,F. Highly significant variations (*p* ≤ 0.001) were found under control, and 10% and 15% PEG treatments for all the 16 days old seedling’s traits (Table 3). The significance and LSD values of ANOVA analysis of control vs. drought treatments for all traits indicated significant variations for all accessions (Table 4).

**Figure 4:**
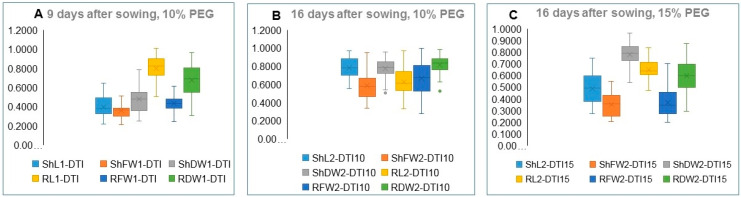
Box and whisker charts showing variation in the DTIs of the seedling traits: (**A**) under 10% PEG for the 9-day-old seedlings, (**B**) under 10% PEG for 16-day-old seedlings and (**C**) under 15% PEG for the 16-day-old seedlings. Full names of shoot traits and their DTIs are in Table 2.

The variation in seedling traits DTIs under 10% PEG for the 9-day-old seedlings and under the 10% and 15% PEG treatments for the 16-day-old seedlings is illustrated in Figure 4 by the lower and upper values of each DTI boxplot. In the 9-day-old seedlings (Figure 4A), the ShL1-DTI, ShFW1-DTI, RFW1-DTI, and ShDW1-DTI were substantially lower than the RL1-DTI and the RDW1-DTI. In the 16-day-old seedlings, the value of the shoot and root traits DTIs under 10% PEG (Figure 4B) were generally higher compared to the corresponding values for the 9-day-old seedlings except for RL2-DTI. The ShDW2-DTI for Zea 3582 and Zea 3244 scored much lower values compared to other accessions and appeared as outliers (Figure 4B). In seedlings exposed to 15% PEG, the DTIs for the examined traits were generally lower compared to seedlings exposed to 10% PEG (Figure 4B,C), but ShDW2-DTI scored higher value compared to the DTIs for other traits. In brief, DTIs for the 16-day-old seedlings exposed to 10% PEG treatment were generally higher than their corresponding values in seedlings exposed to 15% PEG. The range of variation is particularly large for ShFW2, RL2, and RFW2. The DTIs for shoot and root traits of 16-day-old seedlings in all accessions are given in Table A2.

### 3.3. Variation in Leaf Length, Width, and RWC

**Figure 5:**
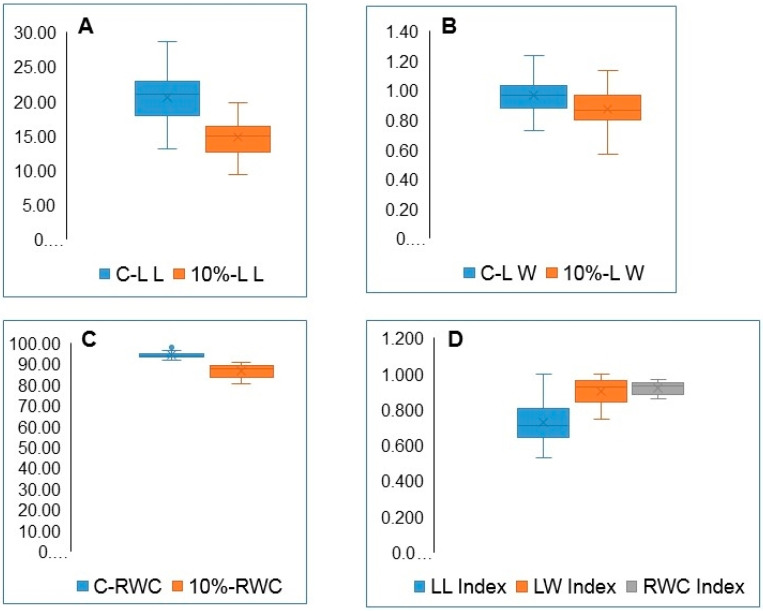
Box and whisker charts showing variation in leaf traits and their DTIs, for 21-day-old seedlings under control condition and 10% PEG treatments. (**A**) leaf length (LL), (**B**) leaf width (LW), (**C**) leaf relative water content (RWC), (**D**) boxplots illustrating the reduction in LL-DTI, LW-DTI, and RWC-DTI by the PEG treatments.

The variation in leaf length and width and in the RWC values are illustrated in Figure 5 for 21-day-old seedlings under normal conditions and 10% PEG treatments. The calculated means for LL, LW, and RWC under the 10% PEG are significantly reduced compared to the control. This is strongly supported by the highly significant values obtained by the ANOVA analysis of data (Table 3) and the LSD values for the control vs. 10% PEG treatment given in Table 4. However, the scale indicating the lower and upper limits of variation in LL and LW boxplots of mean values is greater than the scale for the RWC (Figure 5D). The lower and upper values for each DTI also indicate narrower variation among accessions in the RWC-DTI. It is evident from the values and the boxplots in Figure 5 that LW and RWC have higher DTIs than LL. Values of the DTIs of these three traits in all accessions are given in the Appendix A, Table A3.

### 3.4. Heritability of Traits in Control and PEG-Stressed Traits

The calculated heritability (*H*^2^) values of G% are generally similar under control conditions and the 10% PEG treatment (Table 3). Higher concentrations of PEG drastically reduced the value of G% *H*^2^ from 0.72 for the control to 0.58 for both the 15% and 20% PEG treatments. However, the *H*^2^ of the AS% is low for the control (0.36) and increased as the PEG concentration increased to 0.66, 0.77, and 0.84 under the 10%, 15%, and 20% PEG treatments, respectively. The calculated *H*^2^ values of the shoot and root traits of the 9-day-old and 16-day-old seedlings are generally similar for the control seedlings and seedlings stressed with the 10% PEG treatments. However, particularly low *H*^2^ values are recorded for the ShDW1 (0.28) and RDW1 (0.37) in 9-day-old control seedlings and in seedlings exposed to 10% PEG for ShDW1 (0.43) and RDW1 (0.46). For the 16-day-old seedlings, *H*^2^ values are slightly lower for all traits in seedlings exposed to 15% PEG treatments. The *H*^2^ values of leaf traits are also given in Table 3 and are slightly higher under control conditions compared to the 10% PEG.

### 3.5. Correlations of Traits under Control and PEG Stress 

**Figure 6:**
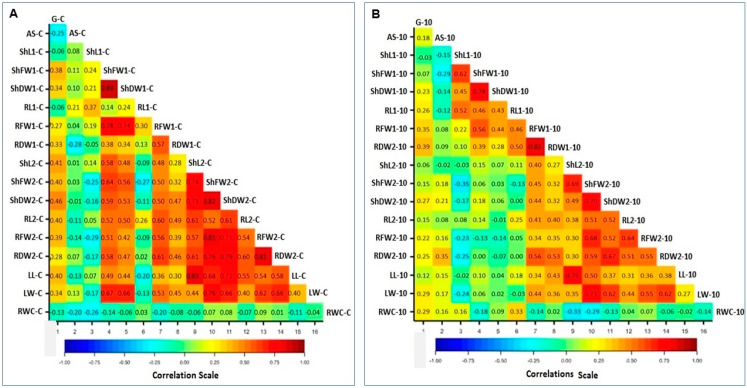
Correlations of 17 traits of maize accessions based on their mean value: (**A**) under control condition, (**B**) under 10% PEG treatment, the correlation coefficient values are plotted on the cells of the correlation triangle produced by GenStat. Names of traits are abbreviated as in Table 2.

Correlations (*r*-value) of the studied 17 traits under control conditions and under the 10% PEG are presented in Figure 6. Under the non-stressed conditions, 14 highly significant *r* values ≥ 0.70 *** have been recorded, 3 for SHFW1, SHDW1 and RFW1 of the 9-day-old seedlings with each other and 11 for the shoot and root traits of the 16-day-old seedlings (ShL2, ShFW2, ShDW2, RL2, RFW2, and RDW2). Additionally, 34 highly significant positive *r* values ≥0.50 *** were scored for ShFW1, ShDW1, RFW1, and RDW1 of the 9-day-old seedlings and all shoot and root traits of the 16-day-old seedlings, except AS and RWC (Figure 6A). The LL and LW are also significantly correlated with the six shoot and root traits of the 16 days old seedlings (RL2, ShFW2, ShDW2, RL2, RFW2, and RDW2). Low *r* values were scored for the control G% and RDW1, while ShFW2 and RFW2 are significantly correlated at the 0.05 significance. On the other hand, the *r* coefficient values are mostly negative or low and insignificant for the traits AS%, ShL1, RL1, and RWC of the control. 

Positive correlations were also scored for the majority of the same 17 traits under the 10% PEG treatment, as indicated by the red and yellow cells in the correlation triangle (Figure 6B). However, the *r* values are generally low compared to their corresponding values under the control condition; only 25 highly significant *r* values are ≥ 0.5 *** for six shoot and root traits of the 9-day-old seedlings. Most of the shoot and root traits of the 16-day-old seedlings, i.e., ShL2, ShFW2, ShDW2, RL2, RFW2, and RDW2, are significantly correlated with each other. The LL and LW are also mostly significantly correlated with each other but at a lower significance level. On the other hand, negative and insignificantly positive *r*-values were recorded between the shoot and root traits for the 9-day-old seedlings and the 16-day-old seedlings and for the G%, AS% and RWC. The RWC is relatively higher correlated with the G% and AS% under the 10% PEG treatment compared to the control conditions (Figure 6A,B).

**Figure 7:**
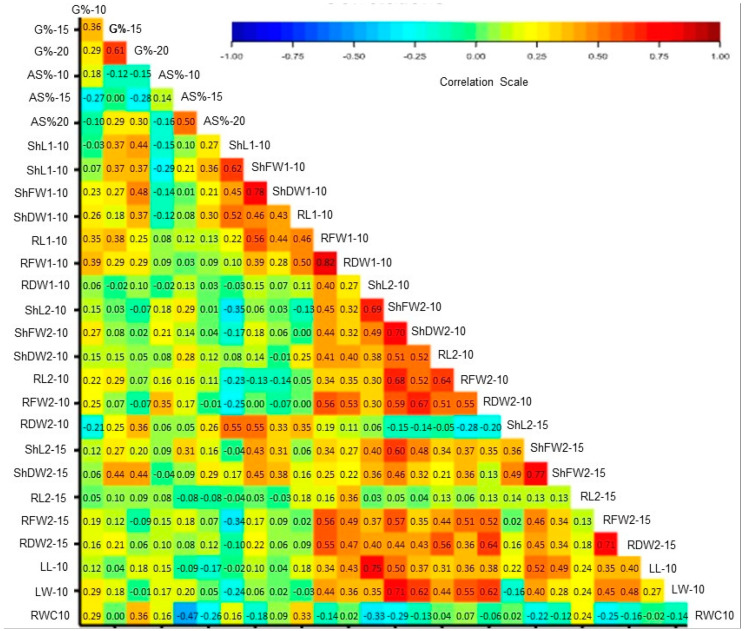
Correlations of 27 traits, including G% and AS% under 10%, 15% and 20% PEG treatments and seedling’s treats under 10% and 15% PEG treatments. Correlation *r*-values are plotted on the cells of the figure produced by GENSTAT. Names of traits are abbreviated as in Table 2.

Correlations of 27 traits, including the above 17 traits, and 10 other traits, including G% and AS% under 15% and 20% PEG treatments and shoot and root traits in seedling exposed to 15% PEG treatments for 16 days, are illustrated in Figure 7. In general, traits of 9-day-old seedlings are often positively correlated with each other but negatively or weakly correlated with traits of the 16-day-old seedlings. Traits of 16-day-old seedlings exposed to 15% PEG, and LL and LW are also often positively correlated with each other. Weak or negative *r*-values are common for the AS% and RWC. Correlation coefficients of 27 germination, seedlings and leaf traits gown under PEG stress treatments are given in Table S1. The correlation of DTIs of 27 traits of maize accessions has been measured and is illustrated in Figure S1. Most of the DTIs of germination and shoot traits of the 9-day-old seedlings are positively correlated with each other and with most shoot and root DTIs of 16-day-old seedlings. The *r* values and significance values for the correlation of traits DTIs are given in Tables S2 and S3. 

### 3.6. Screening for Drought-Tolerant Traits and Accessions

To screen for the most and least tolerant traits and accessions, the frequency of the best performing accessions in the 5% top traits and the least performing accessions in the 5% bottom traits under control and stress treatments is shown in Table 5. Detailed inspection of this table shows that three accessions scored the best performance in ≥10 traits; these are Zea 1062 (16 traits), Zea 3301 (12 traits), and Zea 3602 (16 traits). On the other hand, six accessions scored the least performance in ≥10 traits; these are Zea 323 (13 traits), Zea 633 (24), Zea 677 (17), Zea 3244 (23 traits), Zea 3301 (10 traits), and Zea 3346 (10 traits). The three accessions, Zea 1006, Zea 1019, and Zea 1114, were not among the accessions scoring least performance in the examined traits, whereas, the three accessions, i.e., Zea 12, Zea 242 and Zea 3346 are not among the accessions exhibiting the top mean value of the examined traits (Table 5). Table S2 lists the top 10% accessions scoring best performance estimated as the maximum mean of G% and shoot, root and leaf traits, and minimum AS% under control condition and 10% PEG treatment. Table S3 lists the bottom 10% accessions scoring lowest performance estimated as the minimum mean of G% and shoot, root and leaf traits and maximum AS% under control condition and 10% PEG treatment.

**Figure 8:**
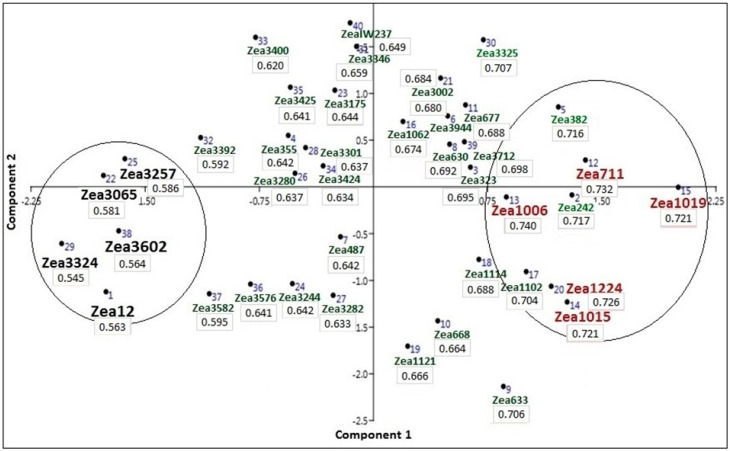
PCA scatter diagram illustrating the grouping of the 40 maize accessions based on the DTI values. The five accessions having the highest DTIs are Zea 711, Zea 1006, Zea 1015, Zea 1019, and Zea 1224 and the five accessions having the least DTIs are Zea 12, Zea 3324, Zea 3602, Zea 3065, and Zea 3257. The DTI value for each accession is given below as the ID in the scatter diagram.

A PCA scatter diagram illustrating the grouping of the 40 maize accessions based on the grand DTI values is shown in Figure 8. The scattering of accessions in arranged from lower DTIs on the negative left side of the *x*-axis to positive DTIs to the right of the *x*-axis of the scatter diagram. The five accessions having the highest grand DTIs are Zea 1006 subsp indurata var. vulgate from Libya (0.74), Zea 711 subsp everata, var. oryzoides from Tshechnia (0.732), Zea 1224 subsp everata var. gracillima from Romania (0.726), Zea 1015 subsp indurata var. vulgate from Libya (0.721), and Zea 1019 subsp everata var. oryzoides from Italy (0.721). Another four accessions scored grand DTI ≥ 0.7; these are Zea 242, Zea 382, Zea 3325, and Zea 1102. On the other hand, the five accessions having the least grand DTI are Zea 3324 from Albania (0.545), subsp indentata; Zea 12 from Germany (0.56), subsp indurata var. vulgata; Zea 3602 from Turkey (0.564), subsp indentata; Zea 3065 from Georgia (0.581), subsp indurata var. alba; Zea 3257 from Albania (0.58), subsp indentata var. xantodon. Also, Zea 3392 and Zea 3582 have grant DTI less than 0.6. The remaining 25 accessions have grand DTI ranging from 0.620 for accession Zea 3400 to 0.698 for Zea 3712 (Figure 8). The display of accessions in the PCA scatter diagram clearly demonstrates the resemblance of accessions having similar DTIs. However, resemblance in DTI values for accessions does not clearly reflect their origin or their assignments to subspecies and varieties as identified in the IPK collection (Table 1).

## 4. Discussion

The performance of maize germplasm for stress-tolerant traits may be best analyzed by effective screening for discriminating between drought-tolerant and drought-susceptible genotypes by easily measured and evaluated traits. The applied drought stress treatments clearly exerted a negative impact on germination and seedling performance of all maize accessions by retarding shoot and root-related traits. Moreover, significant reductions in seedling’s traits under the 10% PEG treatment were evident for all traits, after 9 and 16 days of sowing. Another result that demonstrates the low capacity of maize to tolerate drought stress is the high proportion of abnormal seedlings under the 15% and 20% PEG treatments. The retarded emergence of radicles and plumules of seeds exposed to 15% and 20% PEG and the slow growth of seedlings under these two treatments confirm the view that maize is a drought non-tolerant cereal compared to barley [9] and wheat [10]. It is widely accepted that the first action of moisture deficit imposed by drought is impaired germination, resulting in poor plant stand at the early seedling phase and hampering early crop establishment [8,18,36]. The genetics of germination under abiotic stress is not well understood, but recent studies on the genetic variation for the studied traits by GWAS analysis identified several adaptive genes associated with G% and G%-DTI, on different chromosomes under drought, but no genes were identified for G% under control [9,37].

In maize, as in other cereals, seminal roots are responsible for the initial absorption of moisture and nutrients, but selection for an extended root system reaching larger depths is equally important for efficient acquisition of nutrients [18]. In addition to root characters, drought stress reduces the phenotypic expression of all the seedling traits such as shoot length and the fresh and dry weight of shoot and root [36]. Reduction in seedling growth is the result of restricted cell division and enlargement, as drought stress directly reduces growth by decreasing cell division and elongation [38,39]. Reduction in shoot length is due to less water absorption and a decrease water deficit created by external osmotic potential [36,40]. In cereals, plant growth performance was found to be positively associated with well-developed root systems, as well as early seedling traits [23,27,41,42], both of which can help to improve stress tolerance. However, significant reductions in root length and root fresh and dry mass under simulated drought occurred in most accessions.

The broad-sense heritability (*H*^2^) was estimated under both control and drought conditions. The *H*^2^ of G% was reduced, and *H*^2^ for AS% increased as the PEG concentration increased. The *H*^2^ for seedlings traits are generally similar and values are generally high. However, particularly low *H*^2^ values are recorded for the ShDW1 and RDW1 in the control seedlings and in seedlings exposed to 10% PEG for the 9-day-old seedlings and for the RDW2 in control seedlings and seedlings exposed to 10% and 15% PEG treatments for the 16-day-old seedlings. The higher values of *H*^2^ among traits indicate that selection of maize tolerant genotypes may be based on shoot length and shoot and root fresh and dry weight as well as leaf length and width. Similar heritability values in seedling traits, across nitrogen level applications, were reported in maize, whereas more variation was found in adult plants [32]. This result agrees with the estimates that heritability and genotypic correlation coefficients were significantly high for most of the seedling traits in maize [43].

One important objective of this study is to elucidate correlations of seedling traits with a view to identifying novel traits for measuring drought tolerance at seedling stages among accessions. Comparison of the correlation values under the control condition and the applied stress treatments indicated significantly lower *r*-values of G% and AS% with increased stress levels. However, the *r*-values under stress are generally lower compared to their corresponding values under the control condition, but highly significant *r*-values were scored for most shoot and root trait pairs under 10% PEG stress for the 9-day-old and 16-day-old seedlings. This confirms the view that the effect of stress, as an environmental variable, on the correlations of the studied traits is small [34]. Under the 15% PEG stress, the *r*-value for trait pairs is generally lower compared to the corresponding *r*-values under 10% PEG and the control. At the 15% PEG stress level, RL1 and RL2 were not correlated with other traits. In view of positive correlations of shoot and root trait pairs, it may be concluded that selection for shoot and root weight traits would be effective in identifying genotypes for better performance under moderate drought stress conditions.

In maize, significant negative correlations for seedling traits in early and extra-early maturing maize hybrids were reported [24], particularly for fresh shoot weight, shoot moisture content, root-shoot dry weight ratio, and total fresh biomass. Correlations of seedling traits were also used for selection in wheat and barley [8,9,27]. Phenotypic correlation describes the variance that two traits share based on phenotypic measurements; it includes genetic components that are the proportion of variance that pairs of traits tested share due to genetic factors and environmental correlation imposed by external conditions. The high correlations for shoot and root biomass trait pairs and leaf length and width, recorded in this study, under normal conditions and under stress, indicate that such traits are, to a large extent, genetically controlled. Thus, focusing on these traits would provide information to evaluate genetic variability for seedling traits in maize accessions to effectively screen a large number of accessions in a short period of time.

Another major objective of this study is the classification of maize accessions based on their response to drought stress. The 40 accessions were displayed in a PCA scatter diagram based on the calculated DTI values. The grouping of accessions in the PCA based on the contribution of the DTIs of the examined traits are demonstrated in a PCA biplot, which indicated that the five accessions having highest DTIs and the five accessions having the least DTIs are grouped as two distinct groups from other accessions, as in Figure 8. The most contributing DTIs are those concerned with the shoot and root traits and LL, which are often significantly positively correlated, as indicated in Figure 5. Drought tolerance in maize hybrids has been evaluated using the PCA analysis [29]. Similar results on the selection of drought-tolerant genotypes of durum wheat, based on the combination of indices by the biplot method, were reported [44], thereby this method is better than one index alone to identify superior genotypes for drought conditions. More recently, maize-inbred lines and their hybrid responses to a range of macro and micro-environmental stresses were characterized in terms of water use efficiency (WUE), grain yield, and environmental index [30]. Water use efficiency for drought-tolerant hybrids was significantly greater than for non-drought tolerant hybrids [45].

In the current study, accessions with contrasting response to induced drought stress at the seedling stage (most tolerant vs. most susceptible) can be used for additional experiments to determine how well a seedling‘s drought tolerance can predict the stability of yield under drought in different environments and genetic backgrounds [21,32] in order to identify accessions with potential for higher grain yield for selection of genotypes for breeding commercial lines. For this major objective, evaluation of physiological and biochemical responses are necessary, such as, chlorophyll content photosynthesis rate, chlorophyll fluorescence as well as stomatal conductance, ROS production and osmolytes accumulatiins [46]. In addition, genome-wide association mapping (GWAS) may be applied to identify QTL controlling the variation of traits associated with drought tolerance and seedling development. In this respect, Xu et al. [47] identified candidate genes for drought tolerance in 15 maize inbred lines by whole-genome resequencing. The identification of candidate genes which have roles in the biological pathways of desired traits may be confirmed by finding an association between these trits and their genes by GWAS [48].

## 5. Conclusions

Evaluation of germination and seedling root, shoot and leaf traits were performed under induced osmotic stress simulated by PEG treatments as a profound base for drought tolerance of selected accessions. All PEG treatments significantly reduced germination and retarded seedling early growth; the 15% and 20% PEG treatments resulted in a significant proportion of abnormal seedlings. Positive correlations were found between most trait pairs under control and the 10% PEG treatment, particularly shoot and root traits. Medium to high heritability of shoot and root seedling traits were calculated, providing a sound basis for further genetic analyses. The DTI values were most useful in the differentiation of traits and accessions; PCA analysis based on variation in DTIs clearly grouped the accessions with high DTIs together and the accessions with low DTIs together, indicating resemblance between accessions with similar DTIs. In brief, using seedling traits is a cost-effective approach in achieving rapid screening for tolerant or sensitive maize germplasm in a short time.

## Figures and Tables

**Table 1:** List of the examined Leibniz Institute of Plant Genetics and Crop Plant Research (IPK) maize accession IDs, subspecies, and variety taxonomic information and country of origin.

Serial	Accession ID	Accession Information	Country of Origin
01	Zea 12	subsp indurata (Sturtev) Zhuk. var. vulgata Körn.	Germany
02	Zea 242	subsp indurata (Sturtev) Zhuk. var. vulgata Körn.	Germany
03	Zea 323	subsp everata (Sturtev) Zhuk. var. oryzoides Körn.	Soviet Union
04	Zea 355	subsp saccharata (Körn.) Zhuk var. flavodulcis Körn.	Soviet Union
05	Zea 382	subsp indurata (Sturtev) Zhuk. var. vulgata Körn.	Romania
06	Zea 394	subsp indentata (Sturtev) Zhuk.Ashoro Zairai.	Japan
07	Zea 487	subsp indurata (Sturtev) Zhuk. var. rubropaleata Körn.	Greece
08	Zea 630	subsp indurata (Sturtev) Zhuk. var. vulgata Körn.	China
09	Zea 633	subsp everata (Sturtev) Zhuk. var. oryzoides Körn.	China
10	Zea 668	subsp indentata (Sturtev) Zhuk. var. xantodon Alef.	Macedonia
11	Zea 677	subsp indurata (Sturtev) Zhuk. var. vulgata Körn.	Hungary
12	Zea 711	subsp everata (Sturtev) Zhuk. var. oryzoides Körn.	Tschechnia
13	Zea 1006	subsp indurata (Sturtev) Zhuk. var. vulgata Körn.	Libya
14	Zea 1015	subsp indurata (Sturtev) Zhuk. var. vulgata Körn.	Libya
15	Zea 1019	subsp everata (Sturtev) Zhuk. var. oryzoides Körn.	Italy
16	Zea 1062	subsp indentata (Sturtev) Zhuk. var. flavorubra Körn.	Korea
17	Zea 1102	subsp indurata (Sturtev) Zhuk.	Korea
18	Zea 1114	subsp indurata (Sturtev) Zhuk. Var. aurantiaca.	Italy
19	Zea 1121	subsp indurata (Sturtev) Zhuk.	Austria
20	Zea 1224	subsp everata (Sturtev) Zhuk. var. gracillima Körn.	Rumania
21	Zea 3002	subsp indurata (Sturtev) Zhuk.	Georgia
22	Zea 3065	subsp indentata (Sturtev) Zhuk. var. leucodon Alef.	Georgia
23	Zea 3175	subsp indurata (Sturtev) Zhuk. var. alba Alef.	Georgia
24	Zea 3244	subsp everata (Sturtev) Zhuk. var. oxyornis Körn.	Germany
25	Zea 3257	subsp indentata (Sturtev) Zhuk. var. xantodon Alef.	Albania
26	Zea 3280	subsp indurata (Sturtev) Zhuk. var. vulgata Körn.	Rumania
27	Zea 3282	subsp indurata (Sturtev) Zhuk. Oarzan.	Rumania
28	Zea 3301	subsp indurata (Sturtev) Zhuk. var. vulgata Körn.	Portugal
29	Zea 3324	subsp indentata (Sturtev) Zhuk.	Albania
30	Zea 3325	subsp indentata (Sturtev) Zhuk. var. leucodon Alef.	Albania
31	Zea 3346	subsp indurata (Sturtev) Zhuk. var. caesia Alef.	USA
32	Zea 3392	subsp indurata (Sturtev) Zhuk. var. rubra Bonaf.	Bulgaria
33	Zea 3400	subsp semidentata Kuleshov.	Georgia
34	Zea 3424	subsp indurata (Sturtev) Zhuk. var. rubropalata Körn.	Italy
35	Zea 3425	subsp indurata (Sturtev) Zhuk. var. vulgata Körn.	Germany
36	Zea 3576	subsp indurata (Sturtev) Zhuk. var. alba Alef.	Italy
37	Zea 3582	subsp semidentata Kuleshov.	Koroatia
38	Zea 3602	subsp indentata (Sturtev) Zhuk.	Turkey
39	Zea 3712	subsp everata (Sturtev) Zhuk. var. gracillima Körn.	Georgia
40	IW 237	Cultivar. imported by the Agriculture Research Center	Egypt

**Table 2:** Germination and seedling’s traits description and abbreviations under control and polyethylene glycol (PEG) drought treatments and the drought tolerance indices (DTIs) used to evaluate traits response to drought treatments.

Trait	Abbreviation	Description/Methodology
Germination %	G%	Calculated as G% = n ÷ N × 100, where n is the number of germinated seeds (radicle ≥3 mm) and N is the total number of sown seeds
Abnormal Seedling %	AS%	Seedlings that failed to develop into healthy seedlings after two weeks of sowing
Shoot length	ShL	Maximum length of shoot measured from the point of attachment to grain (cm)
Shoot Fresh weight	ShFW	Weight of fresh shoot detached from the grain and immediately weighed in grams.
Shoot Dry weight	ShDW	Weight of dried fresh shoot put in kraft bag and dried in oven at 70 ± 5 °C for 48 h in grams.
Root length	RL	Maximum length of fresh roots measured from the point of attachment to the grain (cm)
Root Fresh Weight	RFW	Fresh weight of roots of each plant separated from the seed and immediately weighed in grams.
Root Dry Weight	RDW	Weight of dried fresh roots put in kraft bag, dried in oven at 70 ± 5 °C for 48 h for complete drying in grams.
Leaf Length	LL	Fifth leaf length of 21 days old seedlings in cm
Leaf Width	LW	Fifth leaf maximum width of 21 days old seedling in cm
Relative Leaf Water Content	RWC	RWC% calculated as: [(FM − DM)/(TM − DM)] × 100, where, FM, TM and DM, are the fresh, turgid and dry masses respectively of leaf disc weighed using Sartorius Cubis MSU balance in grams.
Germination Drought Tolerance Index	G-DTI	G% under PEG drought/G% under control × 100 G-DTI10%, G-DTI15%, and G-DTI20%
Abnormal Seedlings Drought Tolerance Index	AS-DTI	AS% under PEG drought/AS% under control × 100 AS-DTI10%, AS-DTI15%, and AS-DTI20%,
Shoot Length Drought Tolerance Index	ShL-DTI	ShL under PEG drought/ShL under control × 100 ShL-DTI10% and ShL-DTI15%
Shoot Fresh Weight Tolerance Index	ShFW-DTI	ShFW under PEG drought/ShFW under control × 100 ShFW-DTI10% and ShFW-DTI15%
Shoot Dry Weight Drought Tolerance Index	ShDW-DTI	ShDW under PEG drought/ShDW under control × 100 ShDW-DTI10% and ShDW-DTI15%
Root Length Drought Tolerance Index	RL-DTI	RL under PEG drought/RL under control × 100 RL-DTI10% and RL- RL-DTI15%
Root Fresh Weight Drought Tolerance Index	RFW-DTI	RFW under PEG drought/RFW under control × 100 RFW-DTI10% and RFW-DTI15%
Root Dry Weight Drought Tolerance Index	RDW-DTI	RDW under PEG drought/RDW under control × 100 RDW-DTI10% and RDW-DTI15%
Leaf Length Drought Tolerance Index	LL-DTI10%	LL under PEG drought/LL under control × 100
Leaf Width Drought Tolerance Index	LW-DTI10%	LW under drought/LW under control × 100
Relative Water Content Drought Tolerance Index	RWC-DTI10%	RWC under PEG drought/RWC under control × 100

**Table 3:** Analysis of variance and heritability for the measured maize germination and seedling traits under control and PEG treatments for all accessions. Trait names are as abbreviated in Table 2.

Trait	Control			10% PEG Treatment			15% PEG Treatment			20% PEG Treatment		
	Mean	Acc.	Herit	Mean	Acc.	Herit	Mean	Acc.	Herit	Mean	Acc.	Herit
G%	94.28	***	0.72	90.39	***	0.71	83.86	***	0.58	73.41	***	0.58
AS%	7.41	***	0.76	17.68	***	0.73	32.44	***	0.77	48.68	***	0.84
ShL1	13.09	***	0.91	5.32	***	0.97						
ShFW1	0.550	***	0.86	0.190	***	0.86						
ShDW1	0.056	0.02	0.28	0.189	***	0.43						
RL1	16.34	***	0.60	13.00	***	0.85						
RFW1	0.382	***	0.84	0.189	***	0.89						
RDW1	0.056	0.51	0.37	0.070	0.44	0.46						
ShL2	28.68	***	0.85	22.12	***	0.81	13.86	***	0.78			
ShFW2	0.925	***	0.83	0.529	***	0.82	0.305	***	0.72			
ShDW2	0.084	***	0.79	0.062	***	0.80	0.045	***	0.75			
RL2	27.20	***	0.82	19.87	***	0.78	17.68	***	0.79			
RFW2	0.742	***	0.89	0.485	***	0.82	0.248	***	0.76			
RDW2	0.063	***	0.85	0.052	***	0.82	0.038	***	0.76			
LL	20.63	***	0.79	14.78	***	0.64						
LW	0.954	***	0.85	0.883	***	0.85						
RWC	94.22	***	0.85	86.56	***	0.70						

Acc = accession’s significance; Herit = heritability; *** = high significant (*p* ≤ 0.001).

**Table 4:** Analysis of variance for control/treatment of the measured traits in all accessions. Trait names are abbreviated, as in Table 2.

Trait	Control vs. 10% PEG		Control vs. 15% PEG		Control vs. 20% PEG	
	Significance	LSD	Significance	LSD	Significance	LSD
G%	***	7.350	***	8.98	***	10.08
AS%	***	7.858	***	9.22	***	10.15
ShL1	***	0.226				
ShFW1	***	0.015				
ShDW1	***	0.008				
RL1	***	0.457				
RFW1	***	0.107				
RDW1	***	0.572				
ShL2	***	0.822	***	0.771		
ShFW2	***	0.043	***	0.039		
ShDW2	***	0.010	***	0.009		
RL2	***	0.835	***	0.853		
RFW2	***	0.045	***	0.039		
RDW2	***	0.003	***	0.003		
LL	***	0.618				
LW	***	0.026				
RWC	***	0.562				

*** = high significant (*p* ≤ 0.001).

**Table 5:** Frequency of the best performing accessions in the 5% top traits and least performing accessions in the 5% bottom traits under control and stress treatments (10%, 15%, and 20% PEG).

Serial	Accession ID	Number of Best Traits				Total	Number of Least Traits				Total
		Control	10%	15%	20%	Top 5%	Control	10%	15%	20%	Bottom 5%
1	Zea 12	0	0	0	0	0	0	1	0	0	1
2	Zea 242	0	0	0	0	0	0	0	1	1	2
3	Zea 323	1	1	1	1	4	7	4	2	0	13
4	Zea 355	2	1	1	0	4	2	0	1	1	4
5	Zea 382	0	0	1	0	1	1	0	0	1	2
6	Zea 394	1	6	1	0	8	1	0	0	0	1
7	Zea 487	3	0	0	0	3	1	0	1	0	2
8	Zea 630	1	5	3	0	9	0	1	0	0	1
9	Zea 633	0	0	0	1	1	11	9	4	0	24
10	Zea 668	0	0	2	0	2	1	0	1	0	2
11	Zea 677	0	0	1	0	1	9	6	1	1	17
12	Zea 711	2	0	1	0	3	4	0	0	0	4
13	Zea 1006	2	2	1	0	5	0	0	0	0	0
14	Zea 1015	1	3	0	0	4	0	2	0	0	2
15	Zea 1019	1	2	1	1	5	2	3	3	0	8
16	Zea 1062	5	7	4	0	16	0	0	0	0	0
17	Zea 1102	0	0	1	0	1	1	1	0	0	2
18	Zea 1114	3	3	1	0	7	0	0	0	0	0
19	Zea 1121	3	5	0	1	9	0	1	1	0	2
20	Zea 1224	1	2	2	1	6	2	1	0	1	4
21	Zea 3002	0	0	0	1	1	0	2	0	0	2
22	Zea 3065	3	1	1	0	5	2	2	0	0	4
23	Zea 3175	1	0	0	0	1	1	2	1	0	4
24	Zea 3244	0	0	0	1	1	9	8	5	1	23
25	Zea 3257	1	0	0	0	1	1	1	1	0	3
26	Zea 3280	1	2	2	0	5	0	1	0	0	1
27	Zea 3282	0	0	2	0	2	0	0	3	0	3
28	Zea 3301	8	2	2	0	12	7	2	1	0	10
29	Zea 3324	2	0	0	0	2	0	2	0	0	2
30	Zea 3325	3	3	2	1	9	1	0	0	0	1
31	Zea 3346	0	0	0	0	0	4	5	1	0	10
32	Zea 3392	0	1	0	0	1	0	4	0	0	4
33	Zea 3400	3	6	0	0	9	0	1	0	0	1
34	Zea 3424	2	5	2	0	9	0	0	1	0	1
35	Zea 3425	0	1	0	0	1	1	0	1	1	3
36	Zea 3576	2	1	0	0	3	1	0	3	0	4
37	Zea 3582	0	1	0	0	1	0	2	0	0	2
38	Zea 3602	11	4	1	0	16	0	1	0	0	1
39	Zea 3712	1	2	0	0	3	2	2	0	0	4
40	Zea IW237	0	1	0	0	1	0	1	0	0	1
	Total	64	67	33	7	171	65	65	32	7	173

## Supplementary Materials

The following are available online at https://www.mdpi.com/2223-7747/9/5/565/s1, Figure S1: Correlations of 27 traits DTIs of maize accessions, under PEG stress treatments; the correlation *r* values are plottedon the cells of the correlation triangle produced by GENSTAT and names of traits are abbreviated as a in Table 2. Table S1. Correlation coefficient values of 27 germination, seedlings and leaf traits gown under PEG stress treatments. Table S2. Correlation coefficients of 27 germination, seedlings, and leaf trait’s DTIs for seedlings grown under PEG stress treatments. Table S3. Significance values for the correlation coefficients of 27 germination and seedlings, and leaf traits for seedling under stressed conditions.

## Appendix A

**Table A1:** Drought tolerance indices (DTIs) for germination percentage and abnormal seedlings proportion under 10%, 15%, and 20% PEG treatments, as well as the root and shoot traits of the 9-day-old seedlings for all 40 maize accessions. Names of traits DTIs are abbreviated as in Table 2.

Accession	GI 10%	GI 15%	GI 20%	AS DTI10	AS DTI15	AS DTI20	ShL1 DTI	RL1 DTI	ShFW DTI	RFW DTI	ShDW DTI	RDW DTI
Zea 12	0.98	0.86	0.64	0.86	4.71	4.57	0.40	0.61	0.36	0.60	0.45	0.69
Zea242	0.96	0.97	0.92	0.50	3.50	10.5	0.42	0.82	0.50	0.60	0.56	0.81
Zea323	0.85	0.85	0.85	1.50	8.50	18.0	0.42	0.85	0.51	0.60	0.72	0.76
Zea355	0.98	1.00	0.89	1.00	5.71	6.71	0.24	0.62	0.36	0.59	0.30	0.52
Zea382	0.93	0.93	0.88	1.00	5.00	7.50	0.49	0.79	0.36	0.46	0.51	0.79
Zea394	0.94	0.87	0.81	0.80	3.80	7.60	0.44	0.83	0.36	0.44	0.49	0.85
Zea487	0.93	0.90	0.75	1.00	5.33	5.83	0.41	0.83	0.38	0.45	0.53	0.66
Zea630	1.00	0.93	0.85	4.00	5.50	10.0	0.54	0.93	0.38	0.41	0.53	0.31
Zea633	0.97	0.95	0.84	3.00	4.00	5.00	0.59	0.94	0.51	0.55	0.72	0.93
Zea668	0.91	0.94	0.76	2.00	7.60	7.58	0.53	0.94	0.38	0.40	0.53	0.80
Zea677	0.93	0.91	0.64	1.78	2.89	3.22	0.35	0.56	0.34	0.61	0.42	0.37
Zea711	1.00	0.93	0.69	8.00	27.0	37.0	0.55	1.00	0.49	0.32	0.67	0.69
Zea1006	0.95	0.95	0.90	1.83	2.17	5.33	0.33	0.73	0.33	0.46	0.48	0.88
Zea1015	1.00	0.95	0.86	3.25	3.00	6.75	0.51	0.94	0.38	0.40	0.55	0.85
Zea1019	1.00	0.96	0.98	2.33	2.50	5.00	0.58	0.73	0.50	0.61	0.78	0.77
Zea1062	0.98	0.96	0.86	14.1	23.1	32.1	0.37	0.77	0.30	0.39	0.34	0.53
Zea1102	0.98	0.97	0.86	6.00	12.00	20.0	0.54	0.84	0.38	0.45	0.49	0.95
Zea1114	0.98	0.98	0.72	3.00	8.33	9.33	0.48	0.84	0.40	0.48	0.59	0.81
Zea1121	0.94	0.89	0.86	3.04	6.30	7.41	0.65	1.01	0.39	0.38	0.40	0.92
Zea1224	1.00	0.95	0.93	0.67	1.00	5.00	0.55	1.00	0.45	0.44	0.58	0.96
Zea3002	0.87	0.94	0.74	2.24	3.92	3.14	0.34	0.54	0.36	0.57	0.55	0.63
Zea3065	0.91	0.89	0.78	5.35	4.87	12.0	0.23	0.63	0.21	0.34	0.32	0.41
Zea3175	0.98	0.72	0.73	2.36	2.50	4.23	0.30	0.64	0.29	0.46	0.44	0.81
Zea3244	0.86	0.80	0.64	2.33	2.27	2.80						
Zea3257	1.00	0.95	0.74	2.33	4.33	6.67	0.33	0.50	0.25	0.51	0.35	0.40
Zea3280	0.98	0.93	0.75	3.00	3.00	10.0	0.33	0.75	0.29	0.38	0.38	0.58
Zea3282	0.97	0.78	0.76	2.17	2.00	3.83	0.38	0.85	0.39	0.45	0.49	0.58
Zea3301	1.00	0.72	0.69	2.29	2.57	2.57	0.28	0.90	0.30	0.33	0.43	0.80
Zea3324	0.80	0.78	0.73	2.75	4.10	5.10	0.27	0.63	0.22	0.35	0.32	0.38
Zea3325	0.96	0.91	0.85	1.00	5.00	15.0	0.37	0.82	0.33	0.40	0.48	0.55
Zea3346	0.98	0.75	0.73	2.20	3.80	6.60	0.38	1.00	0.31	0.30	0.25	0.67
Zea3392	1.00	0.93	0.73	4.00	3.40	7.00	0.24	0.63	0.29	0.31	0.30	0.55
Zea3400	0.96	0.91	0.72	3.49	5.66	6.17	0.22	0.84	0.23	0.27	0.33	0.76
Zea3424	1.02	0.88	0.70	1.83	2.44	6.72	0.32	0.78	0.37	0.48	0.52	0.74
Zea3425	0.99	0.81	0.64	1.19	3.50	3.85	0.40	0.75	0.34	0.46	0.47	0.60
Zea3576	0.98	0.85	0.65	6.00	14.0	16.0	0.39	1.00	0.40	0.40	0.57	0.73
Zea3582	1.00	0.81	0.75	6.33	5.33	7.33	0.37	0.75	0.34	0.45	0.70	0.52
Zea3602	0.98	0.89	0.74	5.67	5.67	5.50	0.32	0.78	0.25	0.32	0.36	0.55
Zea3712	0.98	0.81	0.81	2.75	3.75	6.00	0.40	0.85	0.35	0.41	0.47	0.79
ZeaIW237	0.95	0.88	0.75	3.33	6.33	7.67	0.31	0.77	0.29	0.24	0.34	0.58

**Table A2:** Drought tolerance indices (DTIs) calculated for shoot and root traits of 16-day-old seedlings for all 40 maize accessions under 10% and 15% PEG treatments. Names of traits DTIs are abbreviated as in Table 2.

Accession	ShL2 DTI10	ShFW2 DTI10	ShDW2 DTI10	RL2 DTI10	RFW2 DTI10	RDW2 DTI10	ShL2 DTI15	ShFW2 DTI15	ShDW2 DTI15	RL2 DTI15	RFW2 DTI15	RDW2 DTI15
Zea 12	0.80	0.46	0.74	0.40	0.28	0.63	0.32	0.21	0.74	0.63	0.25	0.29
Zea242	0.93	0.63	0.87	0.69	0.67	0.90	0.64	0.47	0.87	0.62	0.33	0.59
Zea323	0.81	0.65	0.71	0.55	0.81	0.73	0.45	0.38	0.71	0.76	0.49	0.56
Zea355	0.75	0.64	0.85	0.61	0.67	0.79	0.46	0.47	0.85	0.69	0.36	0.48
Zea382	0.88	0.85	0.78	0.69	0.84	0.70	0.60	0.48	0.78	0.64	0.46	0.65
Zea394	0.88	0.75	0.80	0.54	0.85	0.70	0.47	0.34	0.80	0.62	0.40	0.56
Zea487	0.66	0.50	0.65	0.67	0.62	0.75	0.44	0.42	0.65	0.64	0.42	0.65
Zea630	0.88	0.65	0.85	0.63	0.69	0.85	0.55	0.55	0.85	0.64	0.34	0.73
Zea633	0.81	0.45	0.77	0.82	0.42	0.91	0.66	0.32	0.77	0.64	0.33	0.44
Zea668	0.82	0.47	0.78	0.74	0.42	0.84	0.61	0.34	0.78	0.56	0.27	0.67
Zea677	0.89	0.66	0.77	0.84	0.63	0.78	0.75	0.51	0.77	0.71	0.70	0.65
Zea711	0.93	0.73	0.69	0.71	0.82	0.90	0.66	0.43	0.69	0.71	0.46	0.79
Zea1006	0.73	0.63	0.96	0.78	0.71	0.98	0.57	0.43	0.96	0.76	0.35	0.70
Zea1015	0.65	0.43	0.88	0.97	0.64	0.90	0.63	0.47	0.88	0.74	0.55	0.76
Zea1019	0.84	0.95	0.89	0.82	0.71	0.91	0.69	0.46	0.89	0.67	0.33	0.59
Zea1062	0.88	0.67	0.80	0.70	0.74	0.84	0.61	0.39	0.80	0.59	0.32	0.61
Zea1102	0.78	0.53	0.82	0.80	0.67	0.85	0.62	0.37	0.82	0.63	0.42	0.83
Zea1114	0.69	0.57	0.86	0.81	0.67	0.77	0.56	0.41	0.86	0.64	0.34	0.63
Zea1121	0.77	0.41	0.87	0.71	0.52	0.76	0.55	0.20	0.87	0.70	0.20	0.43
Zea1224	0.71	0.34	0.85	0.76	0.81	0.94	0.54	0.31	0.85	0.65	0.43	0.85
Zea3002	0.93	0.83	0.80	0.53	0.64	0.76	0.50	0.40	0.80	0.71	0.50	0.88
Zea3065	0.64	0.47	0.85	0.47	0.50	0.87	0.30	0.23	0.85	0.78	0.21	0.55
Zea3175	0.75	0.69	0.79	0.55	0.88	0.80	0.41	0.39	0.79	0.60	0.59	0.42
Zea3244	0.80	0.51	0.76	0.74	0.48	0.53	0.59	0.22	0.76	0.81	0.22	0.40
Zea3257	0.71	0.50	0.66	0.46	0.56	0.86	0.33	0.28	0.66	0.73	0.22	0.55
Zea3280	0.75	0.39	0.75	0.60	0.67	0.86	0.45	0.23	0.75	0.63	0.44	0.77
Zea3282	0.66	0.52	0.79	0.82	0.45	0.70	0.54	0.38	0.79	0.67	0.28	0.54
Zea3301	0.79	0.59	0.82	0.47	0.77	0.89	0.37	0.31	0.82	0.67	0.21	0.62
Zea3324	0.56	0.40	0.78	0.61	0.38	0.79	0.34	0.22	0.78	0.50	0.24	0.54
Zea3325	0.90	0.81	0.81	0.57	0.86	0.93	0.51	0.36	0.81	0.61	0.49	0.80
Zea3346	0.94	0.66	0.83	0.42	0.90	0.77	0.39	0.28	0.83	0.61	0.49	0.70
Zea3392	0.70	0.48	0.70	0.58	0.77	0.87	0.41	0.21	0.70	0.53	0.37	0.68
Zea3400	0.71	0.69	0.86	0.39	1.00	0.83	0.27	0.30	0.86	0.47	0.43	0.41
Zea3424	0.67	0.61	0.54	0.62	0.80	0.82	0.42	0.28	0.62	0.47	0.49	0.56
Zea3425	0.94	0.53	0.92	0.33	0.80	0.82	0.31	0.24	0.92	0.64	0.31	0.56
Zea3576	0.72	0.53	0.71	0.52	0.50	0.86	0.37	0.22	0.71	0.62	0.26	0.43
Zea3582	0.63	0.42	0.50	0.58	0.39	0.79	0.37	0.35	0.60	0.52	0.24	0.60
Zea3602	0.60	0.42	0.54	0.54	0.54	0.87	0.32	0.23	0.54	0.56	0.28	0.42
Zea3712	0.79	0.63	0.72	0.63	0.80	0.96	0.49	0.45	0.72	0.84	0.43	0.62
ZeaIW237	0.97	0.77	0.74	0.51	0.92	0.64	0.49	0.43	0.74	0.73	0.30	0.45

**Table A3:** Drought tolerance indices (DTIs) calculated for leaf length, leaf width, and leaf relative content in 21-day-old seedlings for all 40 maize accessions under 10% PEG treatment.

Accession	LL Index	LW Index	RWC INDEX
Zea 12	0.53	0.79	0.88
Zea242	1.00	0.96	0.87
Zea323	0.78	1.00	0.90
Zea355	0.58	0.84	0.88
Zea382	0.88	0.94	0.90
Zea394	0.77	0.94	0.88
Zea487	0.64	0.93	0.91
Zea630	0.79	0.78	0.94
Zea633	0.66	1.01	0.95
Zea668	0.81	0.77	0.89
Zea677	0.96	0.85	0.88
Zea711	0.81	1.00	0.93
Zea1006	0.74	0.93	0.95
Zea1015	0.68	0.92	0.97
Zea1019	0.96	1.00	0.95
Zea1062	0.80	1.00	0.94
Zea1102	0.57	0.77	0.96
Zea1114	0.72	0.81	0.89
Zea1121	0.73	0.89	0.94
Zea1224	0.65	0.93	0.95
Zea3002	0.83	0.90	0.89
Zea3065	0.59	0.97	0.96
Zea3175	0.60	0.89	0.93
Zea3244	0.71	0.88	0.86
Zea3257	0.65	1.00	0.87
Zea3280	0.75	0.97	0.94
Zea3282	0.69	0.77	0.93
Zea3301	0.65	0.97	0.88
Zea3324	0.55	1.00	0.94
Zea3325	0.92	0.98	0.95
Zea3346	0.70	0.96	0.95
Zea3392	0.56	0.76	0.89
Zea3400	0.63	0.94	0.87
Zea3424	0.67	0.93	0.92
Zea3425	0.84	0.88	0.90
Zea3576	0.56	0.93	0.94
Zea3582	0.70	0.96	0.95
Zea3602	0.69	0.86	0.95
Zea3712	0.90	0.96	0.96
ZeaIW237	0.86	0.95	0.94
